# Metabolic Remodeling during Early Cardiac Lineage Specification of Pluripotent Stem Cells

**DOI:** 10.3390/metabo13101086

**Published:** 2023-10-17

**Authors:** Sunday Ndoma Bobori, Yuxiang Zhu, Alicia Saarinen, Alexis Josephine Liuzzo, Clifford D. L. Folmes

**Affiliations:** Departments of Biochemistry and Molecular Biology and Cardiovascular Medicine, Center for Regenerative Biotherapeutics, Mayo Clinic Arizona, Scottsdale, AZ 85259, USA; bobori.sunday@mayo.edu (S.N.B.);

**Keywords:** metabolomics, induced pluripotent stem cells, 3D cardiomyocyte differentiation, oxidative metabolism, glycolysis

## Abstract

Growing evidence indicates that metabolites and energy metabolism play an active rather than consequential role in regulating cellular fate. Cardiac development requires dramatic metabolic remodeling from relying primarily on glycolysis in pluripotent stem cells (PSCs) to oxidizing a wide array of energy substrates to match the high bioenergetic demands of continuous contraction in the developed heart. However, a detailed analysis of how remodeling of energy metabolism contributes to human cardiac development is lacking. Using dynamic multiple reaction monitoring metabolomics of central carbon metabolism, we evaluated temporal changes in energy metabolism during human PSC 3D cardiac lineage specification. Significant metabolic remodeling occurs during the complete differentiation, yet temporal analysis revealed that most changes occur during transitions from pluripotency to mesoderm (day 1) and mesoderm to early cardiac (day 5), with limited maturation of cardiac metabolism beyond day 5. Real-time metabolic analysis demonstrated that while hPSC cardiomyocytes (hPSC-CM) showed elevated rates of oxidative metabolism compared to PSCs, they still retained high glycolytic rates, confirming an immature metabolic phenotype. These observations support the opportunity to metabolically optimize the differentiation process to support lineage specification and maturation of hPSC-CMs.

## 1. Introduction

Metabolic remodeling is critical during embryonic development to match the constantly evolving catabolic and anabolic demands of the early embryo [1,2]. Not only does metabolic remodeling maintain bioenergetic homeostasis, recent evidence supports the importance of metabolites and energy metabolism in directly regulating cellular fate [3,4]. Indeed, cardiac development and maturation requires dramatic mitochondrial and metabolic remodeling to support the oxidation of a wide array of energy substrates to match the high bioenergetic demands of continuous contraction in the developed heart [5]. Energy metabolism during early development is highly dependent upon substrate supply, with high levels of lactate oxidation supporting the early preimplantation embryo and anaerobic glycolysis predominating in the blastocyst due to an increase in glucose availability and low oxygen environment of the uterus [6]. Following implantation and establishment of blood flow, oxidative metabolism and mitochondria biogenesis and maturation are required to support cardiomyocyte differentiation and maturation [6,7,8]. Nevertheless, anaerobic glycolysis remains a major source of cardiomyocyte ATP production through the perinatal period, accounting for over 40% of ATP production shortly after birth [9]. At birth, circulating levels of oxygen and fatty acids increase, driving further metabolic remodeling and maturation to support high rates of fatty acid oxidation, which becomes the primary source of ATP production for the adult heart [5,9]. 

Although animal models have been utilized to establish this general understanding of metabolic trends in cardiac development, a detailed analysis of how remodeling of energy metabolism contributes to human cardiac development is lacking, in part due to the lack of access to human tissues and challenges of studying metabolism in utero during early development [10]. Differentiation of human pluripotent stem cells (hPSC) into cardiomyocytes offers a tractable system to investigate how metabolic remodeling contributes to human cardiac development [5,11,12]. Similar to the metabolite profile of the late preimplantation blastocysts [13], primed hPSCs primarily rely on glycolysis for energy generation, as well as amino acid metabolism, to generate metabolites for epigenetic regulation of the pluripotent state [2,14]. In vivo differentiation of cardiomyocytes is dependent on the closure of the mitochondrial permeability transition pore to ensure mitochondrial and cardiomyocyte maturation [7,8], which is also observed during in vitro hPSC differentiation as immature round and cristae poor mitochondria of PSCs transition to more mature elongated and cristae rich mitochondria in hPSC-derived cardiomyocytes (hPSC-CMs) [15,16]. Despite mitochondrial remodeling, hPSC-CMs retain immature features that do not recapitulate the features of adult cardiomyocytes, including an immature metabolic phenotype [17,18,19]. Therefore, a major focus of the field has been to metabolically mature differentiated hPSC-CMs by changing metabolite supply, using three-dimensional culture or extended periods of culture [19,20,21,22,23]. While the above studies focus on already specified hPSC-CMs, little is known about how energy metabolism temporally changes and may contribute to early hPSC cardiac lineage commitment. To address this gap in knowledge, we have evaluated temporal changes in energy metabolism during hPSC cardiac lineage specification using three-dimensional differentiation to better recapitulate the in vivo environment.

## 2. Materials and Methods

### 2.1. Human Pluripotent Stem Cell Maintenance as 3D Spheroids

NKX2-5^eGFP/w^ MEL1 human embryonic stem cells were obtained from the iPSC Core Facility at the Murdoch Children’s Research Institute, The Royal Children’s Hospital (NIHhESC-11-0139, David Elliott) [24]. TNNI m-eGFP cells (AICS-0037-172) were generated by the Allen Institute and obtained from the Coriell Institute for Medical Research. hPSCs were maintained in mTeSR™ (Stem Cell Technologies, Vancouver, BC, Canada) or Essential 8 media as 3D spheroids in CELLSPIN spinner flasks (Argos Technologies, Vernon Hills, IL, USA). When spheroids reached a diameter of 250 µm, they were either passaged or induced to differentiate. Spheroids were passaged by dissociation with Accumax supplemented with 220 mU DNAse I and 10 μM Y-27632. Cells were resuspended in media + 10 μM Y-27632 and passed through a 100 µm cell strainer to remove cell aggregates and seeded at 0.5 × 10^6^ cells/mL into fresh flasks. A total of 20 mL of fresh media was added on days 1–3, and 50–70 mL of media changed during subsequent days.

### 2.2. Three-Dimensional Cardiac Differentiation of Human Pluripotent Stem Cells

Cardiac-specific differentiation was induced when spheroids reached an average diameter of 250 µm by treating cells with cell line-specific optimized dose of CHIR99021 (4 µM for TNNI-m-eGFP and 5 µM for NKX2-5^eGFP/w^ MEL1) for 24 h in CDM3 medium, consisting of Roswell Park Memorial Institute (RPMI) 1640 Medium supplemented with 500 µg/mL recombinant human albumin, 210 µg/mL L-ascorbic acid 2-phosphate, and 1X GlutaMAX [25]. Cells were subsequently treated with 2 µM Wnt-C59 in CDM3 medium for 48 h from day 3 to 5. From day 5 onward, media was completely replenished every 2 days. Samples were collected on odd days up to day 15 from 3 independent flasks. GFP expression and beating activity were monitored using an Olympus IX73 fluorescent microscope.

### 2.3. Expression of Lineage Specific Markers by qRT-PCR

Total RNA was extracted from approximately 2 × 10^6^ cells using an RNeasy Plus Mini Kit (Qiagen, Hilden, Germany #74136), and cDNA was synthesized using the iScript Select cDNA Synthesis Kit (BioRad, Hercules, CA, USA #1708897). The cDNA generated was then interrogated via real-time PCR analysis with iTaq Universal SYBR Green Supermix (BioRad, Hercules, CA, USA #1725121) on a BioRad Maestro using primers displayed in Table 1. Results were analyzed utilizing the comparative cycle threshold (ΔΔCt) method normalized to RPS29.

### 2.4. Metabolomics

To ensure consistent cell numbers were collected across time points and flasks, a representative aliquot of cell suspension was harvested, aggregates dissociated with TripLE (day 1–7) or Liberase (day 9–15), and cell numbers were quantified using a Countess Cell counter. Based upon these cell numbers, a volume of cell suspension was harvested corresponding to 5 million cells, centrifuged for 30 s to collect spheroids, washed with 0.9% cold NaCl, and snap frozen in liquid nitrogen. Samples were processed and analyzed in the Mayo Clinic Metabolomics core. Cells were lysed in 1X PBS, and protein was removed by adding 500 μL chilled methanol and acetonitrile solution to cell lysates, followed by lipids removal on Agilent Captiva ND-lipids plates. Cleaned samples were dried down and resuspended in a running buffer prior to LC-MSMS analysis. Central carbon metabolites (219 compounds) were monitored and measured on an Agilent 6460 triple quadrupole mass spectrometer coupled with a 1290 Infinity II quaternary pump [26]. The acquisition was captured in negative electrospray ionization with dynamic multiple reaction monitoring (dMRM) post-ion-pairing reverse phase chromatographic separation. Analytes were searched and confirmed against a curated dMRM database with retention time. Relative abundances between sample sets are derived via multivariate analysis on Agilent Mass Professional Profile (MPP) 14.0 software. Subsequent data analysis was performed with MetaboAnalyst 5.0 [27,28]. Metabolites were filtered out that were not present at a minimum peak height of 50 in at least 2/3 of samples of one experimental group. Data was normalized using sum and log transformations and analyzed with the one factor and metadata tables (for time series data) MetaboAnalyst modules. 

### 2.5. Seahorse Extracellular Flux Analysis

Rates of oxygen consumption (OCR) and extracellular acidification (ECAR) were assessed using a Mitochondrial Stress Test on an Agilent XFe96 Seahorse analyzer. An XFe96 Spheroid Microplate (Agilent) was coated with 40 µL of 1% Fibronectin in dPBS for 1 h at 37 °C. Spheroids were seeded on the coated plate at densities of 10, 20, and 30 spheroids and incubated at 37 °C overnight in CDM3 medium. The following day, media was exchanged for Seahorse XF RPMI media containing 1 mM HEPES (Agilent Technologies, Santa Clara, CA, USA) and supplemented with 2 mM glutamine and 11 mM glucose. Indices of mitochondria function were assessed by sequential addition of 1 µg/mL Oligomycin, 1 µM FCCP, and 1 µM Rotenone/Antimycin A to quantify rates of basal/maximal OCR and spare respiratory capacity. Assay results were normalized to total protein content quantified using a Pierce™ Coomassie (Bradford) Protein Assay Kit (Pierce Biotechnology, Rockford, IL, USA) following lysis with M-PER™ Mammalian Protein Extraction Reagent (Pierce Biotechnology, Rockford, IL, USA). 

### 2.6. Statistical Analysis

Data analysis was conducted using GraphPad prism Version 8 (GraphPad Software, LLC). All data are represented as mean ± SEM with a *p*-value of <0.05 determined to be statistically significant using statistical tests defined in the figure legend.

## 3. Results

### 3.1. Three-Dimensional Differentiation of Human Pluripotent Stem Cells

**Figure 1:**
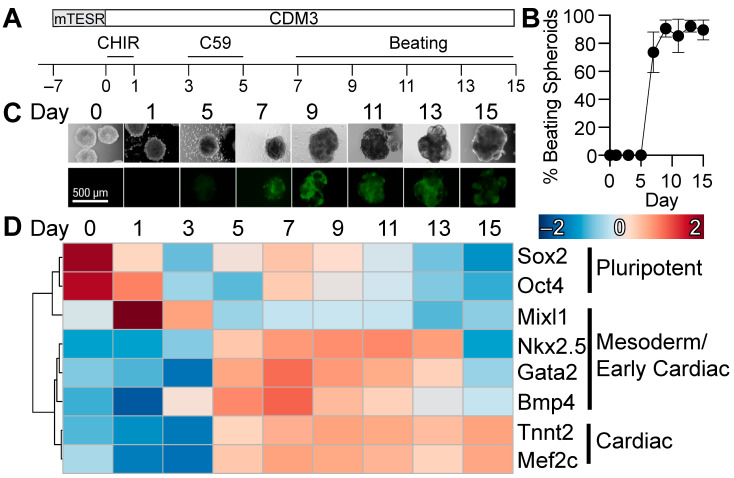
Three-dimensional cardiac-specific differentiation of human pluripotent stem cells. (**A**) Cardiac differentiation scheme including time of small molecule administration and sample collection. (**B**) Percentage of beating spheroids during differentiation. (**C**) Morphological changes during differentiation with the onset of expression of GFP-tagged cardiac NKX 2.5 between days 5 and 7 of differentiation. (**D**) qRT-PCR gene expression analysis of pluripotent, mesoderm, and cardiac lineages demonstrates the onset of cardiac specification as early as day 5 of differentiation. *n* = 3 independent differentiation flasks. Values represent mean ± SEM.

We adapted our established two-dimensional cardiac differentiation protocol [17,25] to facilitate cardiac differentiation in a three-dimensional culture system. Three-dimensional hPSC spheroids were differentiated toward a cardiac cell fate by temporal regulation of Wnt signaling using CHIR99021 (24 h, day 0) and Wnt-C59 (48 h, day 3) (Figure 1A). Induction of cardiac lineage specification was visually monitored by assessing spontaneous contraction, which was observed as early as day 6 of differentiation, with over 70% of spheroids beating 7 days after induction (Figure 1B) and expression of the NKX2.5-GFP reporter, which was faintly induced at day 5 and increased over the remainder of differentiation (Figure 1C). Gene expression analysis of representative lineage-specific genes indicated a rapid downregulation of pluripotency genes Oct4 and Sox2 on day 1 of differentiation, coinciding with expression of mesodermal genes from days 1 to 3 (Mixl1). Early cardiac genes are initially expressed on day 5, consistent with the expression of the NKX2.5-GFP reporter, with peak expression observed between days 7 and 9, while mature cardiac markers (Tnnt2, Mef2c) continue to increase through day 15 of differentiation. Therefore, the 3D differentiation protocol supports the generation of spontaneously contracting cardiomyocyte spheroids with similar temporal changes in lineage-specific genes, as reported in our two-dimensional protocol [17]. 

### 3.2. Intracellular Metabolic Remodeling during hPSC-CM Specification

**Figure 2:**
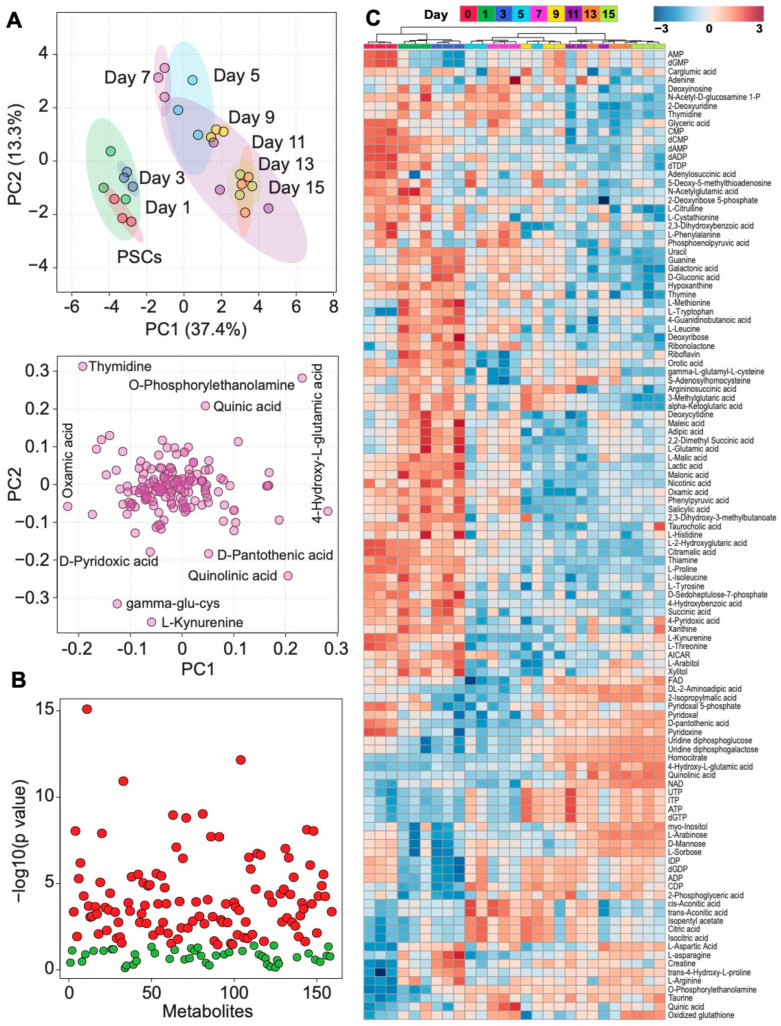
Metabolomics analysis of central carbon metabolism remodeling during three-dimensional cardiac lineage specification. (**A**) Principal component analysis of intracellular metabolites displays sequential segregation of each day of differentiation (top panel) and metabolites responsible for group segregation (loading plot, bottom panel). (**B**) One-way ANOVA and a Fisher’s least significant difference method post hoc analysis identified 114 metabolites that significantly differed over the course of differentiation (red represents FDR corrected *p* < 0.05. *n* = 3, green represents not significant). (**C**) Heatmap showing relative concentrations of 114 significantly different metabolites over the course of cardiac differentiation.

To investigate how energy metabolism is remodeled during PSC-cardiomyocyte lineage commitment, we utilized an LC-MS dMRM approach to quantify metabolites within central carbon metabolism across cardiac differentiation. Principal component analysis (PCA) of metabolite profiles demonstrated temporal metabolic remodeling, with samples clustering into two distinct groups, consisting of days 0–3 (pluripotency and early mesoderm) versus days 5–15 (cardiac specification/maturation) (Figure 2A top panel). Some of the major metabolites responsible for segregation of clusters in the PCA analysis included thymidine, O-phosphorylethanolamine, L-kynurenine, 4-hydroxyglutamate, quinolinic acid, pantothenate, and gamma-glutamylcysteine (Figure 2A bottom panel), which are part of amino acid metabolism, nucleic acid metabolism, NAD^+^ synthesis, phospholipid synthesis, and glutathione metabolism. Of the 159 intracellular metabolites that were consistently detected across all time points, 114 metabolites significantly differed over the course of the differentiation, with relative concentrations observed in the heatmap (Figure 2B,C and Table S1). 

**Figure 3:**
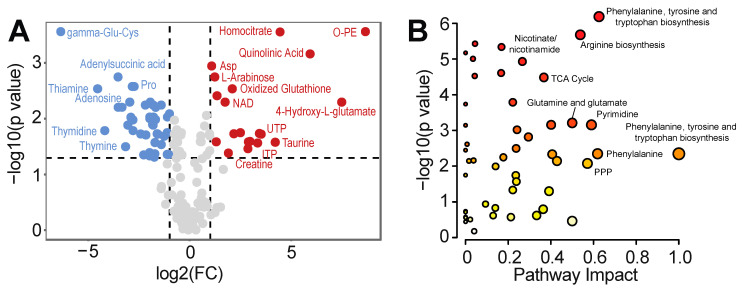
Comparison of metabolic profiles of human pluripotent stem cells vs. day 15 of differentiation. (**A**) Volcano plot identifying differential metabolites between day 0 (PSCs) and 15 of differentiation, using an FDR-corrected *p*-value of 0.05 and a fold-change cutoff of 2. (**B**) Quantitative pathway analysis (Global Test) displaying *p* values of the pathway enrichment analysis versus pathway impact comparing hPSCs (day 0) and day 15 of differentiation.

To identify the metabolic remodeling that occurs during complete cardiac differentiation, we directly compare the metabolic profiles of PSCs (day 0) vs. day 15 of differentiation. A total of 63 metabolites met the criteria of a *p*-value cutoff of 0.05 (FDR corrected) and a fold-change of 2, with 41 metabolites displaying lower and 22 displaying higher abundance on day 15 vs. day 0 (Figure 3A and Table S2). Metabolites upregulated on day 15 included o-phosphorylethanolamine, oxidized glutathione, creatine, homocitrate, arabinose, NAD, and ADP (Figure 3A). Downregulated metabolites included gamma-glutamylcysteine, thiamine, thymine, adenylosuccinic acid, proline, and thymidine (Figure 3A). Quantitative pathway analysis of these metabolites demonstrated over enrichment within pathways associated with amino acid metabolism, pyrimidine metabolism, pentose phosphate pathway, and TCA cycle (Figure 3B and Table S3), consistent with previous observations that directed cardiac differentiation requires a transition from anabolism supporting high rates of proliferation in PSCs to catabolism in support of the energy demanding process of contraction in cardiomyocytes [15]. 

### 3.3. Temporal Analysis Indicates Stalled Metabolic Maturation following Cardiac Lineage Specification

**Figure 4:**
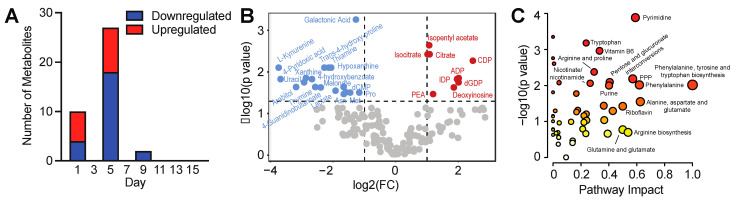
Pairwise day-to-day metabolic remodeling during 3D cardiac differentiation. (**A**) Paired day-to-day comparisons identify critical early time points for metabolic remodeling. (**B**) Volcano plot identifying differential metabolites between days 3 and 5 of differentiation, using an FDR-corrected *p*-value of 0.05 and a fold-change cutoff of 2. (**C**) Quantitative pathway analysis (Global Test) displaying *p* values of the pathway enrichment analysis versus pathway impact from a pathway topology analysis comparing days 3 and 5 of differentiation.

To identify critical time points where metabolic remodeling occurs during differentiation, we performed a pairwise analysis of consecutive time points. We observed that most metabolite abundance changes (FDR corrected *p*-value of 0.05 and minimum 2-fold change) occurred early during differentiation from day 0 to 1 during loss of pluripotency and induction of mesoderm and from day 3 to 5 during the transition from mesoderm to early cardiac (Figure 4A and Table S4). Few changes in metabolite abundance were observed following day 5 of differentiation. We found this to be consistent with PCA analysis revealing a distinct segregation between day 3 and day 5 metabolites on PC1 and PC2 (Figure 2A). Although the PCA plot suggests that day 5 appears to be intermediate between days 7 and 9, pairwise analysis of day 5 vs. 7 and day 5 vs. 9 revealed no significant differences in metabolite abundances. Indeed, we observed spread in the replicate data at day 5, which may be due to the heterogeneity of cell types, including endodermal, endothelial, and cardiac cell types, that appear during the early cardiac/cardiac progenitor stage [29]. We next evaluated up- vs. down-regulated metabolites along with their representative metabolic pathways in day 5 vs. day 3 cells. Metabolites that were upregulated on day 5 included o-phosphorylethanolamine, nucleotides (ADP, dGDP, CDP, IDP, deoxyinosine), TCA cycle intermediates (isocitrate, and citrate) (Figure 4B and Table S5), and creatine, whereas metabolites that were downregulated included lactate, kynurenine, amino acids (aspartate, proline, methionine), purine derivatives (xanthine, hypoxanthine), vitamin B metabolites (thiamine, thymine, 4-pyridoxic acid) (Figure 4B). Quantitative pathways enrichment analysis identified highly enriched pathways, including purine/pyrimidine metabolism, amino acid metabolism (arginine, proline, phenylalanine, tyrosine, and tryptophan), pentose phosphate pathway (PPP), nicotinamide, vitamin B metabolism, and purine metabolism (Figure 4C and Table S6), suggesting a transition from amino acid and nucleic acid synthesis towards increased energy production and phospholipid synthesis during early cardiac specification of PSCs.

**Figure 5:**
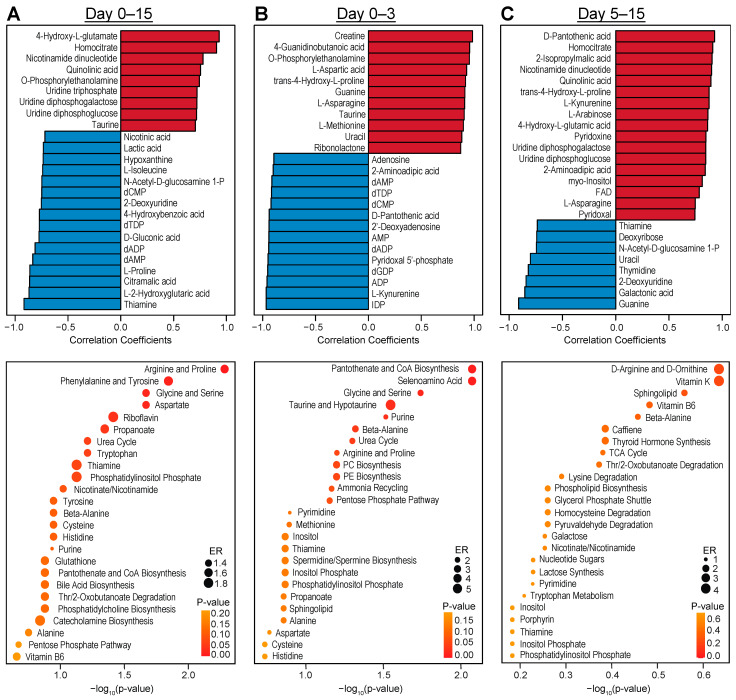
Temporal metabolic analysis indicates that metabolic remodeling is stalled following cardiac specification of hPSCs. Correlation analysis of top 25 metabolites associated with time and Metabolite Set Enrichment Analysis (MSEA) of significantly associated metabolites using linear models with covariate adjustments during the complete time course: days 0–15 (**A**), days 0–3 (**B**), and days 5–15 (**C**).

Metabolites could also progressively change over longer periods rather than the dramatic changes between time points; therefore, we performed temporal correlation analysis across distinct days of cardiac differentiation to identify the metabolites that were most correlated with time. We specifically selected the complete time course (days 0–15, Figure 5A and Table S7), days 0–3 (Figure 5B), and days 5–15 (Figure 5C), guided by the PCA analysis results showing significant variance between day 0 through 3 vs. days 5 through 15 metabolite profiles (Figure 2A). Many of the metabolites most correlated with the overall time course were also identified by the volcano plot in the pairwise comparison between PSCs and day 15 of differentiation, such as increasing abundance of O-phosphorylethanolamine, 4-hydroxy-L-glutamine and homocitrate, and decreasing abundance of thiamine, proline and lactic acid (Figure 5A). We identified the metabolites that are significantly associated with time using linear models with covariate adjustments (Table S7), which confirmed the metabolites shown in the correlation analysis, and used these metabolites to perform Metabolite Set Enrichment Analysis (MSEA), confirming several of the pathways in the PSC vs. day 15 analysis. Analysis of the first three days of differentiation encompassing the transition from pluripotency to mesoderm demonstrates the remodeling of metabolites associated with amino acid, nucleotide, and phospholipid metabolism (Figure 5B). Although, a number of metabolites were associated with time from day 5 to 15 of differentiation (Figure 5C), no pathways were significantly enriched using MSEA, confirming limited metabolic remodeling following lineage specification of hPSCs. 

### 3.4. Assessment of Real-Time Metabolic Function in hPSC-CM

**Figure 6:**
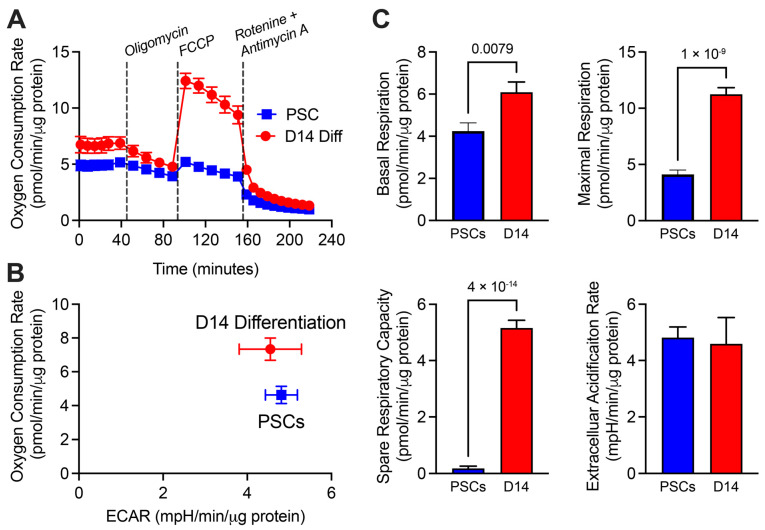
Seahorse extracellular flux analysis of oxygen consumption rates (OCR) and extracellular acidification rates (ECAR). (**A**) Mitochondrial stress test displaying OCR in Day 14 spheroids vs. hPSCs. (**B**) Energy map of relative utilization of OCR vs. ECAR. (**C**) Quantitation of basal respiration, maximal respiration, spare respiratory capacity, and ECAR. *n* = 12, values are mean ± SEM, and *p*-values are displayed based on Unpaired two-tailed *t*-tests.

To functionally assess metabolism in real-time, we performed extracellular flux analysis of PSC spheroids versus day 14 of differentiation, which largely consist of cardiomyocytes (Figure 6 and Figure S1). As expected, day 14 spheroids showed a significant increase in mitochondria oxygen consumption rate (OCR) when compared to parental PSC spheroids, including increases in basal, maximal, and spare respiratory capacity (Figure 6). However, day 14 spheroids also retain high extracellular acidification rates (ECAR), indicating the maintenance of a high glycolytic rate even after specification to a cardiac cell fate, consistent with a lack of a metabolic switch from glycolysis to oxidative metabolism during maturation of PSC-CMs.

## 4. Discussion

A critical regulator of hPSC fate decisions during embryonic development and stem cell lineage specification is energy metabolism. While studies in animals models have demonstrated significant cardiac metabolic remodeling during end-stage development and early postnatal life [30,31,32,33], the mechanistic understanding of metabolic regulation of earlier developmental events is still largely inferred from genetic loss of function studies and metabolic gene expression due to challenges in assessing metabolism in a tissue-specific manner in utero. Ethical concerns with using human fetal tissues and lack of access to human tissues during development further complicate the investigation of metabolic processes during human development [10]. To address these limitations, we have adaapted a monolayer cardiac differentiation protocol to support three-dimensional differentiation of hPSCs into self-organizing cardiac spheroids/organoids that have been demonstrated to display tissue complexity, spatiotemporal organization, and functional characteristics reflective of the developing heart [11,12,34]. This tractable system enabled the tracking of metabolic remodeling across early lineage transitions from pluripotency (day 0) to mesoderm (day 1–3) to cardiac specification (day 5 onward). Using this 3D differentiation, we found significant metabolic remodeling with over 2/3 of detected metabolites displaying differential abundances (Figure 2C) across cardiac lineage specification. Direct comparison of metabolic profiles of day 15 hPSC-CMs to parental hPSCs identified metabolites and pathways previously associated with hPSC-CM differentiation, including increased lipid and mitochondrial energy metabolism following cardiac lineage commitment at the expense of nucleic acid and amino acid metabolism [15,35,36,37,38]. However, when we temporally dissected the differentiation process, we observed that the majority of metabolic changes occurred early during differentiation (days 0 vs day 1 and days 3 vs day 5) associated with transitions between pluripotency and mesoderm, and mesoderm and early cardiac, with only limited metabolic remodeling following day 5 of differentiation associated with cardiac differentiation/maturation. 

Differentiation of PSCs into cardiomyocytes is a highly dynamic process requiring multiple lineage transitions as they become increasingly specialized. Thus, there is a need to identify how and when metabolic remodeling supports lineage specification [16]. Herein, we found that the majority of metabolic remodeling is observed during the early phases of differentiation encompassing mesoderm and early cardiac induction (days 0–5), with limited metabolic remodeling following cardiac lineage specification on day 5. Specifically, we observed 27 metabolites that significantly differed on day 3 vs. day 5 during the transition from mesoderm into early cardiac lineages, encompassing a transition from amino acid and nucleic acid synthesis towards energy production and phospholipid synthesis. Although the role of many of these metabolites has not been directly characterized during cardiac differentiation, potential roles can be hypothesized from other model systems. For example, kynurenine and quinolinic acid are key metabolites in the kynurenine pathway of NAD^+^ synthesis [39], and genetic mutations within this pathway (HAAO or 3-hydroxyanthranilic acid 3,4-dioxygenase and KYNU or kynureninase) resulted in NAD-associated congenital heart defects, highlighting the importance of kynurenine metabolism/NAD^+^ synthesis in cardiogenesis [39]. Moreover, a recent study reports that kynurenine is essential for neonatal heart regeneration and angiogenesis [40]. Recent work has demonstrated temporal requirements for NAD^+^ synthesis during PSC-CM differentiation, with inhibition leading to loss of cell viability and energy generation during early differentiation (prior to day 5) and resistance to NAD^+^ depletion increasing with length of time in culture up to day 28 of differentiation [41]. Vitamin B metabolites (thiamine, thymine, 4-pyridoxic acid) also play a critical role in regulating energy metabolism [42], which can support lineage specification and differentiation, while clinical evidence suggests that thiamine deficiency may lead to cardiac disease, including congestive heart failure and beriberi heart disease [43,44,45,46]. Proline has been shown to support both pluripotency and differentiation by protecting cells from oxidative stress, increasing cytoskeletal flexibility, and serving as an energy source, all of which can be critical during cardiac differentiation by detoxifying reactive oxygen species created by active mitochondria and supporting necessary contractile ability [47,48,49,50]. Therefore, future studies are required to elucidate the molecular mechanisms by which these metabolites regulate cardiac lineage specification. 

PSCs and cardiomyocytes have distinct metabolic requirements, with PSCs requiring carbon biomass and energy to match biosynthetic demands of proliferation and to safeguard their genome, whereas cardiomyocytes require efficient catabolism to match the high energetic demands of continuous contraction [2,14]. Cardiac lineage specification thus requires significant metabolic remodeling from predominantly glycolysis in PSCs to oxidative metabolism in cardiomyocytes. Consistent with these observations, our pathway enrichment analysis of day 0 vs. day 15 confirmed an increase in oxidative stress (increased GSSG) and energy requirement (increased NAD, ADP) at the cost of decreased DNA/RNA synthesis (decreased nicotamide and purine metabolism). Glutathione provides the reducing power for handling intracellular oxidative stress and recent studies show that redox balance maintenance, which is evaluated by the classic GSH/GSSG ratio, is critical in cardiac function and protection [51,52]. While we observed a functional increase in oxidative metabolism with extracellular flux analysis, day 14 spheroids displayed equivalent rates of glycolysis to PSC spheroids despite previous reports that three-dimensional differentiation facilitates more rapid cardiomyocyte maturation [11,53,54]. Indeed, a number of studies have acknowledged the immaturity of PSC-CMs as demonstrated by weaker contractile force, immature electrophysiologic properties, and persistent reliance on glycolysis [16,36,55,56,57,58,59]. It has been suggested that this continued reliance on glycolysis in PSC-CMs may be due to the glucose-rich media with limited oxidizable substrates these cells are typically cultured in, so efforts have focused on promoting metabolic maturation through fatty acid supplementation/media optimization [20,60,61] or pharmacological manipulation of fatty acid oxidation and glucose oxidation [22,62], as well as long-term culture [11,21,23] to promote hPSC-CM maturation. 

Observations that transplantation of early hPSC-CMs (day 5–7) into neonatal rat hearts displayed significantly better maturation compared with late hPSC-CMs (after day 14) suggest that optimal maturation capacity may be dependent on a critical early window during in vitro cardiomyogenesis [63,64,65]. Our previous work demonstrated that cardiomyocyte differentiation using standard in vitro conditions uncoupled hPSC-CM developmental stage from proliferative capacity when compared to normal murine development, including a premature exit from the cell cycle despite having a lineage gene expression profile equivalent to ∼E14.5 mouse cardiomyocytes [17], which are still proliferative in vivo [16]. These observations, paired with the limited metabolite remodeling following cardiac specification herein, suggest that there may be a mismatch between the PSC-CM developmental stage and their in vitro metabolic environment and that earlier metabolic intervention may be required to fully unlock hPSC-CM differentiation and maturation. Indeed, a cluster of TCA cycle intermediates, including cis/trans-aconitic acid, citric acid, and isocitric acid, appear to peak between days 5 and 9 of differentiation and decrease in subsequent days (Figure 2C). Commonly utilized cell culture media does not recapitulate the in vivo metabolic environment found during development [66,67,68], and the limitations of these conditions are well recognized as isolated adult cardiomyocytes rapidly lose their mature phenotype or die when placed in culture [69,70,71,72]. Such environments often have supraphysiologic glucose and oxygen levels while lacking essential metabolites such as fatty acids, diverging from the natural metabolic landscape of an in vivo developing heart [5]. In addition, it is well established that the metabolic environment is constantly changing during normal development and that metabolites and their metabolism are driving forces behind lineage specification and patterning of the embryo [1,73]. This contrasts with the largely static metabolic environment that is used in cell culture and offers the opportunity to investigate whether providing developmentally relevant metabolites facilitates specific stages of differentiation and production of hPSC-CMs. The continued development of physiologically relevant media [66,74,75] will facilitate the investigation of how physiologic metabolite concentrations may support robust PSC-CM differentiation and maturation as well as provide insight into the mechanisms of how the microenvironment reshapes cellular metabolism. This could lead to a more robust in vitro model enabling the investigation of how mitochondria and energy metabolism contribute to normal and abnormal cardiac development. 

## Figures and Tables

**Table 1:** Reverse and forward primer sequences for RT-PCR.

Gene	Reverse	Forward
MEF2C	5′-CCC AAG GAC TAA TCT GAT CGG-3′	5′-CTT TCT CTT TCC TGT TTC CTC CA-3′
NKX 2.5	5′-CAC TCA GCA TTT GTA GAA AGT CAG-3′	5′-ACC CTA GAG CCG AAA AGA AAG-3′
TNNT2	5′-TCT TCG TCC TCT CTC CAG TC-3′	5′-AGA AGA GGT GGT GGA AGA GTA-3′
RPS29	5′-AAT ATG TGC CGC CAG TGT TT-3′	5′-CCC GGA TAA TCC TCT GAA GG-3′
GATA2	5′-CTG TCT GCA ACG CCT GTG-3′	5′-GTT CCG AGT CTG GAT CCC TT-3′
BMP4	5′-GCA CTG GTC TTG AGT ATC CTG-3′	5′-TGC TGA GGT TAA AGA GGA AAC G-3′
OCT4	5′-AGT TTG TGC CAG GGT TTT TG-3′	5′-ACT TCA CCT TCC CTC CAA CC-3′
SOX2	5′-CTT GAC CAC CGA ACC CAT-3′	5′-GTA CAA CTC CAT GAC CAG CTC-3′
MIXL1	5′-GAA GGA TTT CCC ACT CTG ACG-3′	5′-GTA CCC CGA CAT CCA CTT G-3′

## Data Availability

The data presented in this study will be openly available in Metabolomics Workbench upon publication.

## Supplementary Materials

The following supporting information can be downloaded at https://www.mdpi.com/article/10.3390/metabo13101086/s1, Table S1: Differential metabolites across cardiac lineage specification using one-way and a Fisher’s LSD post hoc test.; Table S2: Volcano plot of differential metabolites between PSCs and day 15 of cardiac differentiation.; Table S3: Quantitative pathway analysis of PSCs vs. day 15 of cardiac differentiation.; Table S4: Pairwise day-to-day metabolic remodeling during PSC cardiac differentiation.; Table S5: Volcano plot of differential metabolites between day 3 and day 5 of cardiac differentiation.; Table S6: Quantitative pathway analysis of day 3 vs. 5 of cardiac differentiation.; Table S7: Temporal correlation analysis of metabolites across time points; Figure S1: Individual Seahorse extracellular flux analysis traces.
